# Table of Urinary Deposits

**Published:** 1855-03

**Authors:** 


*Table of Urinary Deposits*, with their microscopical tests, for clinical examinations. By John King, M. D., Cincinnati, Ohio.

For daily referrence in qualitative analysis of the urine, the above table will be found quite convenient. It contains a number of well executed drawings, and to the beginner in the study of the urine, it will prove a valuable and reliable guide.

